# Water-Transfer Slows Aging in *Saccharomyces cerevisiae*

**DOI:** 10.1371/journal.pone.0148650

**Published:** 2016-02-10

**Authors:** Aviv Cohen, Esther Weindling, Efrat Rabinovich, Iftach Nachman, Shai Fuchs, Silvia Chuartzman, Lihi Gal, Maya Schuldiner, Shoshana Bar-Nun

**Affiliations:** 1 Department of Biochemistry and Molecular Biology, George S. Wise Faculty of Life Sciences, Tel Aviv University, Tel Aviv, Israel; 2 Department of Molecular Genetics, Weizmann Institute of Science, Rehovot, Israel; Tulane University Health Sciences Center, UNITED STATES

## Abstract

Transferring *Saccharomyces cerevisiae* cells to water is known to extend their lifespan. However, it is unclear whether this lifespan extension is due to slowing the aging process or merely keeping old yeast alive. Here we show that in water-transferred yeast, the toxicity of polyQ proteins is decreased and the aging biomarker 47Q aggregates at a reduced rate and to a lesser extent. These beneficial effects of water-transfer could not be reproduced by diluting the growth medium and depended on *de novo* protein synthesis and proteasomes levels. Interestingly, we found that upon water-transfer 27 proteins are downregulated, 4 proteins are upregulated and 81 proteins change their intracellular localization, hinting at an active genetic program enabling the lifespan extension. Furthermore, the aging-related deterioration of the heat shock response (HSR), the unfolded protein response (UPR) and the endoplasmic reticulum-associated protein degradation (ERAD), was largely prevented in water-transferred yeast, as the activities of these proteostatic network pathways remained nearly as robust as in young yeast. The characteristics of young yeast that are actively maintained upon water-transfer indicate that the extended lifespan is the outcome of slowing the rate of the aging process.

## Introduction

Increased human life expectancy, with its impact on aging and age-related diseases such as cancer, diabetes, cardiovascular and neurodegenerative diseases, drives extensive ongoing aging research [1]. Studies focus on revealing the basis underlying organismal aging, setting measurable hallmarks of aging, as well as on slowing the aging process as a mean to extend healthspan [2]. Recent aging research is mainly advanced by uncovering aging-related genetic and biochemical pathways that are conserved in evolution and may modify the rate and progression of the aging process [3]. Yet, wide gaps exist in our understanding of aging, when it begins, what drives it, and which biological aspects are affected [4]. This is because multiple and diverse processes affect, and are impacted by, aging. Therefore, various approaches must be taken in studying biological aging [5]. Hayflick's discovery that cells senesce and stop dividing after a limited number of divisions [6], has led to the realization that aging is not merely an organismal phenomenon but also occurs at the cellular level. Hence, aging research can be conducted in cultured cells as well as in unicellular organisms such as yeast.

Yeast and other organisms have evolved to survive under adverse conditions frequently encountered in the wild, such as scarcity of food sources. Under starvation, organisms enter a stationary phase, maintain low metabolism and exhibit extended lifespan and enhanced resistance to stress [7]. Caloric restriction (CR), which was shown to promote longevity in many organisms, extends yeast chronological lifespan (CLS) as well as replicative lifespan (RLS) [7,8], the two primary ways to query the lifespan and aging of *Saccharomyces cerevisiae* [9,10]. Most strikingly, Granot and Snyder demonstrated already more than two decades ago that transfer of *S*. *cerevisiae* from growth medium to pure water (hereafter referred to as 'water-transfer') extended the yeast lifespan [11]. This transfer, which can be regarded as 'extreme CR' [9], more than doubled the CLS of different yeast strains [12–14]. Over the years, this intriguing phenomenon was not fully explored, although several studies have shed some light on its molecular basis. For example, it was shown that the serine/threonine kinase Rim15 and other members of the RAS and TOR pathways were required for yeast CLS extension upon water-transfer [15]. Five VPS genes and three ESCRT components, two of which function in endocytosis and fusion of multivesicular bodies (MVBs) with the vacuole, were also implicated in CLS extension [16]. Additionally, autophagy was upregulated by water-transfer, as well as by CR, and was required for CLS extension upon water-transfer [14].

Most of the above-cited studies deduced aging rate from CLS measurements, overlooking other characteristics of the aging process, especially in response to CR or water-transfer. For example, the sudden redox collapse, which affects more than 80% of thiol-containing proteins, is now regarded as a characteristic of the aging yeast [13]. This redox collapse is slowed by CR and appears to stop upon water-transfer [13]. Similarly, levels of NAD^+^, a key factor in redox homeostasis and in Sir2 functions, drop in aging yeast, and exogenous NAD^+^ precursors extend lifespan, depending on CR [17,18]. Combined, these effects likely reflect low metabolism of yeast under CR or water-transfer, which, in turn, may slow the aging process. In fact, the “wear and tear” theory of aging attributes aging-related damages of DNA and proteins to high rate of metabolism and vigorous cellular activities, generating free radicals [6,19]. Other theories suggest that aging is the consequence of balancing reproduction and proteostasis, so CR and water-transfer can slow aging by shutting down reproduction in response to scarcity of nutrients. However, such hypotheses suggest a passive extension of lifespan awarded by the mere reduction in metabolism, whereas a more interesting prospect is that lifespan extension is a result of an active rewiring of cellular responses under such conditions.

Here we follow the effects of water-transfer on *S*. *cerevisiae* aging by employing, as aging readouts, our recently described aggregation assays [20]. In these assays we have shown that the midsize polyQ protein, 47Q, is soluble in young logarithmically growing yeast but progressively aggregates along yeast aging, reflecting aging-dependent aggregation [20]. In the current study we combined the 47Q aggregation assays with measurements of lifespan and activities of proteostasis pathways and with systematic analysis of protein-level changes. We show that a genetic program, activated upon water-transfer, extends yeast lifespan by slowing the aging process, as reflected by maintaining characteristics of young yeast.

## Materials and Methods

### Yeast strains and plasmids

The *S*. *cerevisiae* wild-type strains were W303-1b (*MATα ura3-1 trp1-Δ2 leu2-3*,*112 his3-11 ade2-1 can1-100*) and BY4741 (*MAT*a *his3Δ1 leu2Δ0 met15Δ0 ura3Δ0*). The polyQ constructs (generously provided by Prof. M. Sherman, Boston University), which contained GFP, were induced by galactose, and referred to as 25Q, 47Q and 103Q, reflecting the length of their polyQ tracts, were previously described [20]. The CPY*-HA (*prc1-1* allele) was expressed from pBG15 plasmid (generously provided by Prof. A. Cooper, Garvan Institute of Medical Research), and the 6myc-Hmg2 was expressed from pRH244 plasmid (generously provided by Prof. R. Hampton, UC San Diego). The heat shock response (HSR) was monitored with HSE2-*lacZ* (GA1695; [21]; generously provided by Prof. I. Dawes, University of New South Wales), having a synthetic HSE2 (ctaGAAgcTTCtaGAAgcTTCtagaggatccccg). The unfolded protein response (UPR) was monitored with UPRE-*lacZ* (pMCZ-Y; generously provided by Prof. R. Schekman, Berkeley University), having a consensus UPRE (tcgaGGAACTGGACAGCGTGTCGAAA) [22]. The 25Q, 47Q, 103Q, HSE-*lacZ* and UPRE-*lacZ* plasmids were of high copy number (2μ), the CPY*-HA and the 6myc-Hmg2 plasmids were centromeric, and all plasmids contained *URA3* as their selectable marker.

### Growth conditions and water-transfer

Yeast was grown in synthetic complete (SC) minimal media described in S1 Table that contained 0.67% (w/v) yeast nitrogen base, 2% (w/v) glucose. For selection of transformants, uracil was eliminated from the SC, and for screening the MATa XXX-GFP::HIS3 library, histidine was eliminated from the SC (S1 Table). For galactose induction, the glucose was replaced by 4% (w/v) galactose. Cells were grown at 30°C for the indicated number of days in 10–30 ml medium in 100 ml loosely-capped bottles with constant shaking (200 rpm). Unless indicated otherwise, water-transfer was usually done after ∼12–20 hours of growth in SC medium, as cells approach the end of logarithmic growth (0.5–0.9 A_600_). Cells were pelleted by centrifugation (18,000 x *g*, 1 minute, 4°C), washed once in an equal volume of sterile double distilled water and resuspended to the original volume of water.

### Viability assay by colony forming units spots and by propidium iodide flow cytometry

To determine yeast viability/survival by colony forming units (CFU) spots, yeast was spotted on yeast extract peptone dextrose (YPD) agar plates at 10-fold serial dilutions in water, starting at 0.5 A_600_ (∼0.75x10^7^ cells). Plates were incubated at 30°C for 2 days. Viability, alone or in combination with polyQ toxicity, was similarly determined but the cells were spotted on SC agar plates supplemented with either 2% (w/v) glucose or 4% (w/v) galactose, as indicated.

Assessment of CLS using propidium iodide (PI, Sigma) was performed as described [23] with minor modifications. At the indicated time points, cells were harvested by centrifugation (18,000 x *g*, 1 minute), resuspended in 1 ml phosphate-buffered saline (PBS), and incubated for 20–50 minutes at 30°C in the presence of 2 μM PI. Approximately 10,000 cells were analyzed by fluorescence-activated cell sorting (FACS calibur; BD sciences, CA, USA), with excitation at 488 nm and emission at 533 nm. Flowing Software (v 2.5.1; www.flowingsoftware.com) was used for data analysis.

### Quantitative fluorescence microscopy

Yeast expressing GFP-tagged polyQ proteins were viewed and analyzed by quantitative fluorescence microscopy, as previously described [20]. Images of hundreds of cells were taken and processed using custom Matlab code [24]. Total fluorescence densities and maximal density of the brightest foci were used to calculate the *R* ratio as a measure for aggregation, as previously described [20].

### Sample collection, alkaline lysis, filter retardation and blotting assays

Yeast samples were collected and cell density was determined at A_600_. Identical number of cells were collected by centrifugation (18,000 x *g*, 1 minute, 4°C), washed in 10 mM NaN_3_ in PBS, and frozen (-20°C), to allow simultaneous processing. Cells were lysed in 0.4 ml /2 A_600_ of lysis buffer (0.2 M NaOH / 71 mM β-mercaptoethanol) followed by incubation on ice for 30 minutes, adjustment to pH 7 with HCl, and boiling, as described [20]. Boiled lysates were adsorbed to, or filtered through 0.2 μm nitrocellulose membranes, in the absence or presence of 2% SDS, respectively. PolyQ proteins and actin were detected by immunoblotting with rabbit anti-GFP antibody (ab290, Abcam) and mouse anti-actin antibody (ab3280, Abcam), respectively, followed by DyLight 680-labled goat anti-rabbit IgG (072-06-15-06, KPL) and IRDye 800CW-conjugated goat anti-mouse antibody IgG (LI-COR Biosciences), respectively. Secondary antibodies were visualized by the Odyssey Infrared Imaging System (LI-COR Biosciences) and quantified by the Odyssey software (v 3.0). Aggregation Indexes were calculated, as described [20].

### Screen procedures

The screen was performed using the MATa XXX-GFP::HIS3 5,330 strain collection [25]. Using a RoToR bench-top colony arrayer (Singer Instruments) the library was refreshed and broken down from stock 1536 format to fresh YPD agar plates (Singer) at 384 format and grown over night at 30°C. The library was then transferred from agar plates into 50 μl SC-His with 2% glucose in 384-well polystyrene flat-bottom plates (Greiner) using the RoTor arrayer and grown overnight at 30°C. Using a Janus Liquid handling robot (Perkin Elmer), 10 μl of cells in SC-His with 2% glucose were transferred to 384-well polystyrene flat-bottom plates pre-filled with 90 μl of water, mixed by pipetting and placed with lid in an incubator (Liconic) for over night incubation at 30°C.

Cells were shaken on day 1 after transfer to water (Janus, 300 rpm) and on the 30 days that followed (tabletop shaker, 900 rpm). The library was imaged after 24 hours (day 1) in 384-well glass bottom MicroWell plates (Matrical). Microscopic screening was performed using an automated microscopy set-up as previously described [26,27] relying on an automated inverted fluorescent microscopic ScanR system (Olympus). Images were acquired using a 60X air lens with 490/20 nm and emission at 528/38 nm (GFP). After imaging, plates were sealed with an AeraSeal breathable sealing film (Excel Scientific) and placed in a plate shaker (Heidolph Inkubator 1000) for 31 days. Plastic seals were then pealed and imaging protocol was repeated as described on day 1. After acquisition, images were manually reviewed using the ScanR analysis program and compared to images taken from logarithmically growing yeast cells in SC-His with 2% glucose.

### Degradation of ERAD substrates

Degradation of the endoplasmic reticulum (ER)-associated protein degradation (ERAD) substrates CPY*-HA and 6myc-Hmg2 was monitored by cycloheximide (CHX) chase, as previously described [28]. Samples were collected, resolved by 10% SDS-PAGE, and immunoblotted with mouse anti-HA antibody (clone 12CA5) and mouse anti-myc antibody (clone 9E10), respectively. Primary antibodies were followed by IRDye 800CW-conjugated goat anti-mouse antibody IgG (LI-COR Biosciences), which was visualized by the Odyssey Infrared Imaging System (LI-COR Biosciences) and quantified by the Odyssey software (v 3.0).

### Responses to heat shock (HSR) and unfolded proteins (UPR)

The HSR was monitored in yeast expressing HSE2-*lacZ* by exposure to 42°C for 20 minutes and the UPR was monitored in yeast expressing UPRE-*lacZ* by exposure to either tunicamycin (5 μg/ml) or dithiothreitol (6 mM) for 1 hour, as previously described [29,30]. These responses were measured as β-galactosidase specific activity.

### β-galactosidase activity

This enzymatic assay was previously described in details [29], monitoring hydrolysis of *ortho*-nitrophenyl-β-galactoside to *ortho*-nitrophenol (ONP) at 420 nm by Genesys 10UV spectrophotometer. Protein concentration was determined with Bradford reagent, using bovine serum albumin as a standard. Specific β-galactosidase activity was calculated as nmol ONP / min / mg protein.

### Statistical analyses

In most measurements and unless specifically noted, each data point represents 2–3 biological replicates, and 3 technical replicates within each biological replicate. Standard error of the mean (SE) over the 6–9 replicates was used to estimate the error at each data point. Standard deviation (SD) was used to measure the quantitative fluorescence variability among 40–250 single cells at each data point.

## Results

### Water-transfer extends yeast lifespan, relieves polyQ toxicity and stalls 47Q aggregation

**Fig 1:**
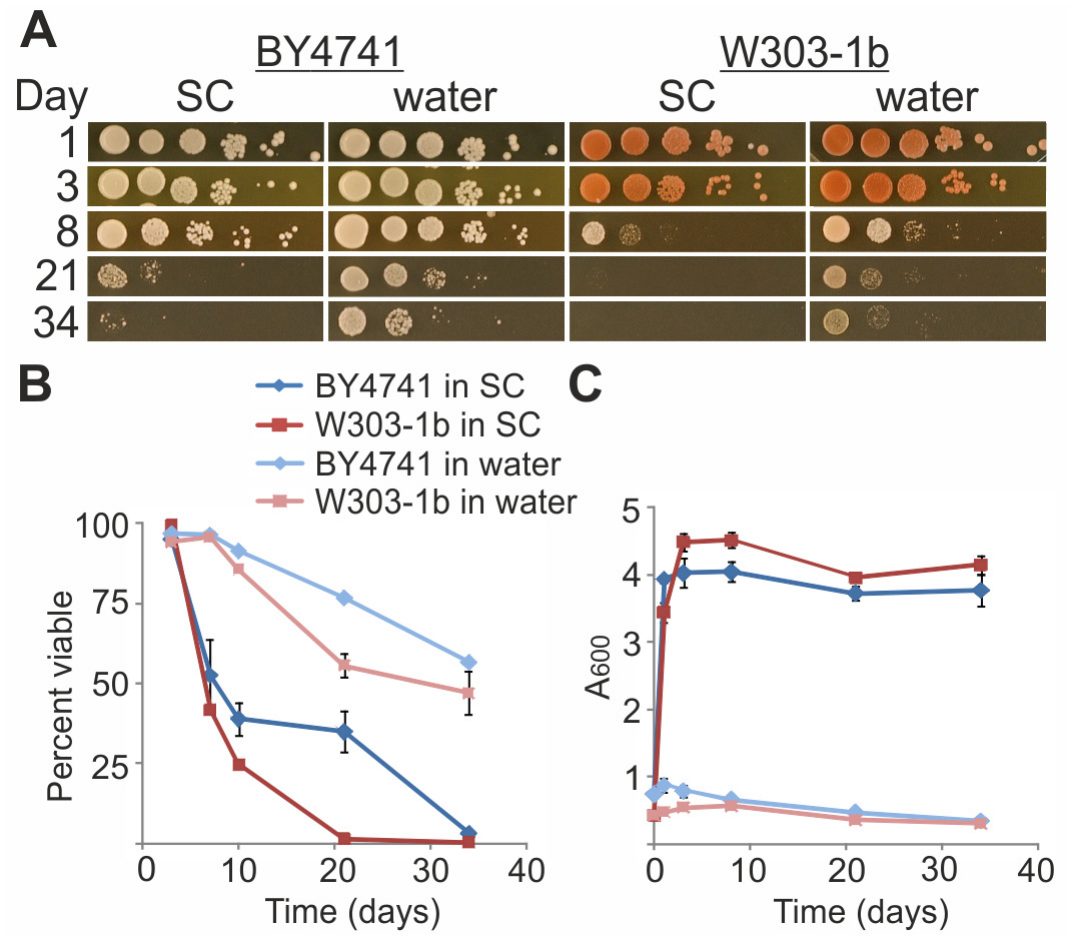
Water-transfer extends yeast lifespan. Yeast strains W303-1b or BY4741 grown in SC with 2% glucose were either maintained in SC with glucose or transferred to water at logarithmic growth (0.5–0.9 A_600_). Cells were collected on the indicated days and analyzed. (A) For CFU viability test, 10-fold serial dilutions (starting with 7.5x10^6^ cells) were spotted on YPD plates. (B) Viability by PI exclusion was monitored by FACS. (C) Growth curves were monitored as A_600_. Data are presented as mean ± SE of 3 biological replicates and technical triplicates within each biological replicate.

Yeast CLS is determined by changes in CFU along aging, followed either by counting individual colonies [10], or by spotting 10-fold serial dilutions (hereafter referred to as ‘CFU spots’; for instance, see [12]). Yeast viability may also be assessed by using the nucleus staining dye propidium iodide (PI), which allows calculating the percentage of live (dye excluders) and dead (fluorescent) cells in a given population [23]. We monitored the effect of water-transfer on yeast survival by counting CFU spots (Fig 1A) and by PI exclusion (Fig 1B). Wild-type yeast strains (W303-1b and BY4741) survived in water for a much longer time than cells grown in SC media (Fig 1A and 1B). These results are in full agreement with previous reports [12,14,31,32]. Strain W303-1b died faster than strain BY4741, either in SC medium or in water (Fig 1A and 1B), in accordance with the notion that lifespan is governed by genetic parameters [31,33]. It should be noted that yeast of both genetic backgrounds stopped dividing immediately upon water-transfer (Fig 1C).

**Fig 2:**
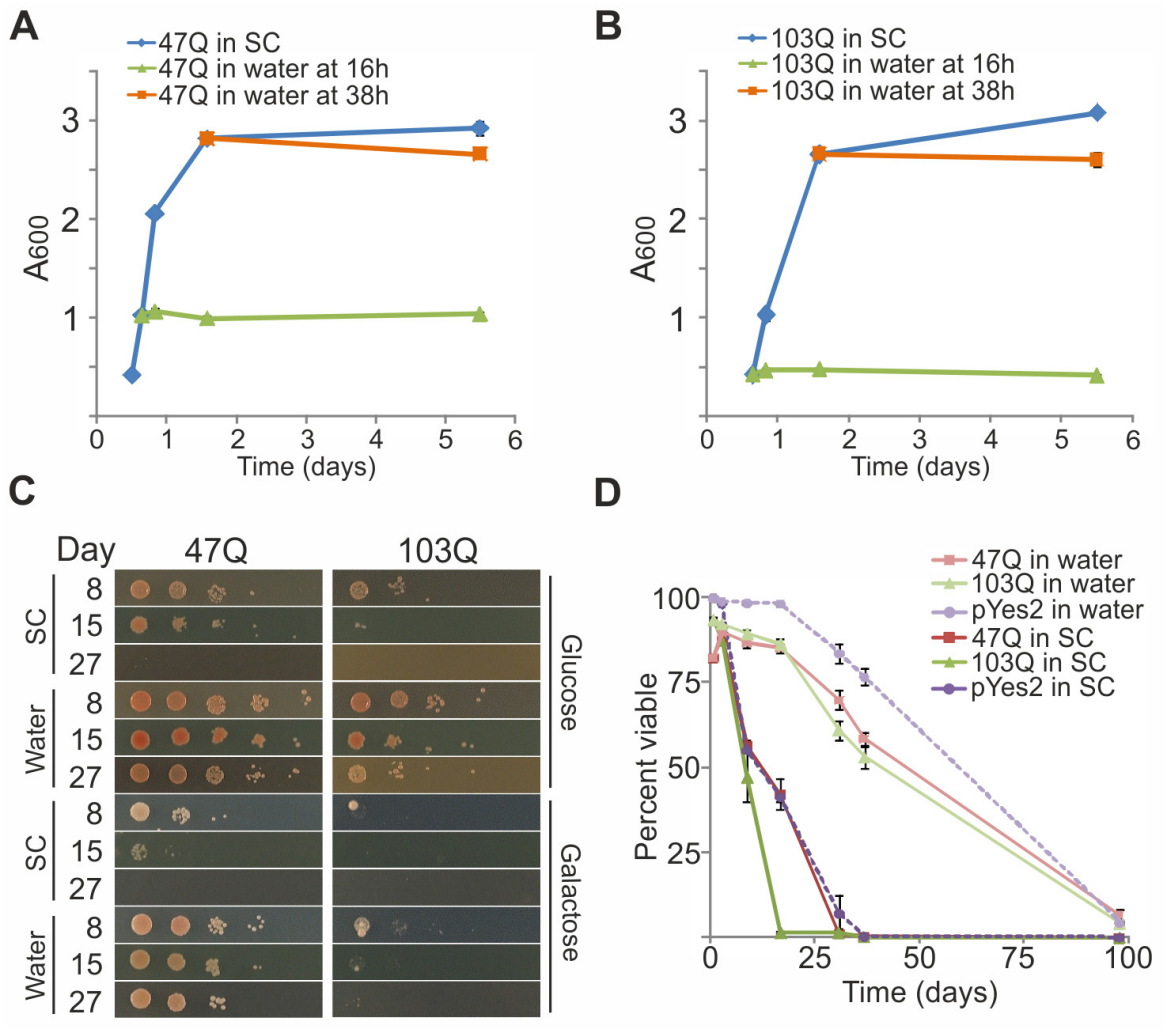
Water-transfer relieves toxicity of polyQ proteins. W303-1b cells expressing the indicated polyQ proteins (47Q, 103Q or the empty vector pYES2) were grown in SC-Ura under galactose induction. At the indicated time points, cells were either transferred to water or maintained in SC-Ura with galactose. On the indicated days cells were collected and analyzed. Growth curves of cells expressing 47Q (A) or 103Q (B) presented as A_600_ are the mean ± SE of 3 biological replicates and technical triplicates within each biological replicate. (C) Cells expressing 47Q or 103Q were transferred to water at logarithmic growth and on the indicated days afterwards were tested for viability by CFU spots on glucose or galactose plates, as described in Fig 1A. (D) Viability by PI staining was monitored as described in Fig 1B. Data from 2 biological replicates and technical triplicates within each biological replicate, are presented as mean ± SE.

We have recently shown that aggregation and toxicity of polyQ proteins, which depend on the length of their polyQ tract, is also affected by yeast aging [20]. Therefore, we next examined the effect of water-transfer on yeast expressing the 47Q or 103Q constructs [20]. As shown in Fig 1C for the untransformed W303-1b cells, yeast harboring the 47Q (Fig 2A) or the 103Q (Fig 2B) proteins also stopped dividing upon water-transfer. Although in most reports yeast was transferred to water at the end of the diauxic shift on day 2 or 3 [34], we found that the optimal transfer time to achieve the full effects of water-transfer was around 16–20 hours of growth in SC with galactose, to induce 47Q or 103Q expression, while approaching the end of the logarithmic phase (∼0.8A_600_). As shown by survival on glucose or galactose plates (Fig 2C), this time window significantly reduced the toxicity of the 47Q and especially of the highly toxic 103Q. Also, by comparing PI exclusion of cells expressing 47Q, 103Q or an empty vector, it was evident that lifespan was similar in all water-transferred cells, which was extended more than 3-fold compared to cells maintained in SC, irrespective of the polyQ construct (Fig 2D). These results also demonstrated that the extreme toxicity of 103Q was relieved upon water-transfer (Fig 2D); the small drop in viability of cells harboring 47Q or 103Q occurred prior to their transfer to water, reflecting the toxicity of these proteins, which was relieved in water (Fig 2D).

Since the polyQ proteins were induced by galactose, it could be argued that the reduced toxicity in water-transferred cells resulted from eliminating these proteins once the inducer was removed. To address this possibility, cells were visualized by fluorescence microscopy at different time points, in order to detect the polyQ proteins via their GFP tag. The polyQ proteins declined in cells that were kept in SC medium supplemented with galactose, whereas the surviving water-transferred yeast contained higher amounts of polyQ proteins that were maintained up to 70 days post-induction (data not shown). Since this difference was evident even for the toxic 103Q, it further supported our observation that the toxicity of polyQ proteins was relieved upon water-transfer (Fig 2D).

**Fig 3:**
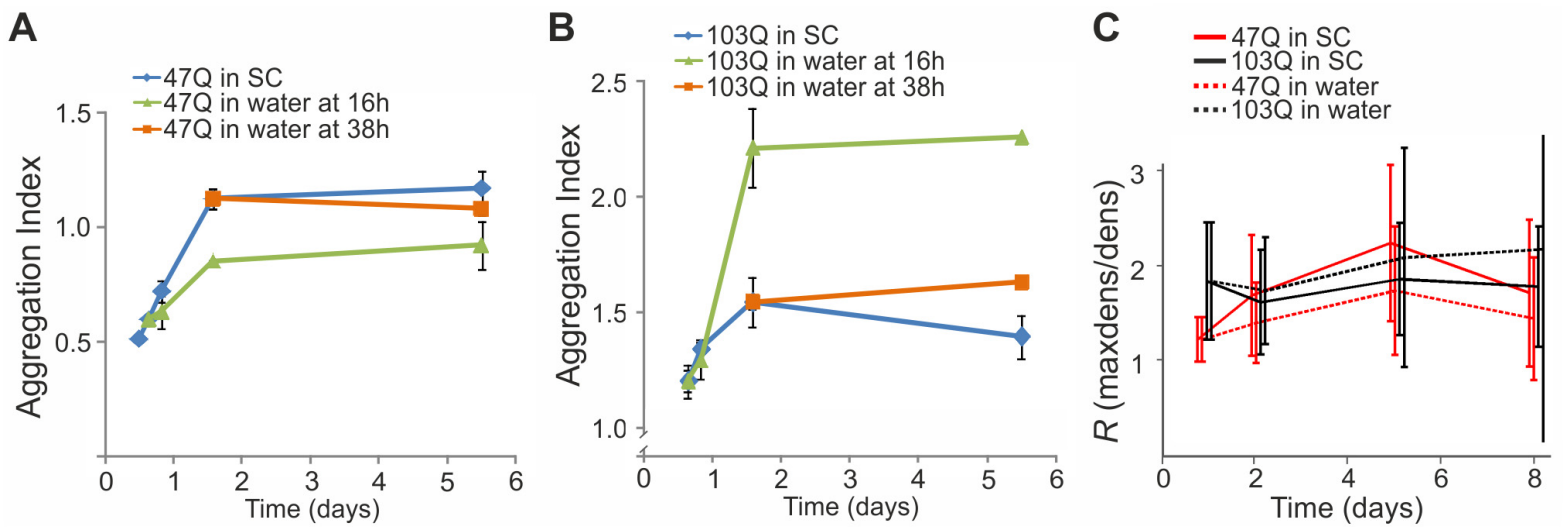
Water-transfer stalls 47Q aggregation. W303-1b cells expressing 47Q (A) or 103Q (B) grown in SC-Ura under galactose induction were treated and collected as in Fig 2A and 2B, to analyze and calculate the Aggregation Index as described in Materials and Methods. Data from 3 biological replicates and technical triplicates within each biological replicate, are presented as mean ± SE. (C) W303-1b cells expressing the indicated polyQ proteins grown in SC-Ura under galactose induction were either maintained in the same medium or transferred to water on Day 1 (logarithmic growth). On the indicated days afterwards, aggregation levels, given as the ratio R, were calculated by fluorescence imaging, as described in Materials and Methods. At each time point the mean values ± SD are shown and the error bars reflect the variability between individual cells. The 47Q shows significant difference between treatments from day 2 and on (P<0.001, two-sided t-test). The large variability in 103Q aggregation under water-transfer conditions results in insignificant differences from both 103Q under SC or 47Q under water conditions in terms of the mean. However, the increased variability is significant (P<0.00002, chi-squared test).

We have previously shown that the aggregation of the mid-size 47Q is exacerbated as the yeast ages, and thus affords a reliable readout for the aging process [20]. Here we followed the aggregation of the polyQ proteins to examine whether the extended lifespan in water was also manifested by changes in their aggregation that likely reflected the rate of the aging process. Aggregation of 47Q and 103Q in cells transferred to water after growing for different periods of time in SC medium with galactose was monitored by either filter retardation assay or the quantitative fluorescence aggregation assay [20]. Opposite aggregation trends were observed for 47Q and 103Q upon transfer to water. As indicated by the Aggregation Index, when cells were transferred to water at their logarithmic growth, 47Q aggregation ceased and reached a lower plateau (Fig 3A), whereas 103Q aggregation proceeded and reached a higher plateau (Fig 3B). Similar results were observed by the quantitative fluorescence measurements, where early transfer to water led to decreased 47Q aggregation but a wider spread in 103Q aggregation levels (Fig 3C). The opposite aggregation trends of 47Q and 103Q may suggest two distinct forms of aggregates, whose handling is processed independently by different mechanisms. Regardless, these results are consistent with the notion that 47Q aggregation depends on aging, since 47Q does not aggregate in log phase cells but progressively aggregates along yeast aging [20]. Conversely, 103Q aggregation is aging-independent but time-dependent, since 103Q aggregates as soon as it is formed [20] and continues to aggregate in water-transferred yeast. More importantly, since 47Q aggregation is an aging readout, its stalling suggests that aging is slowed upon water-transfer.

### The effects of water-transfer on aggregation and toxicity cannot be reproduced by medium dilution

**Fig 4:**
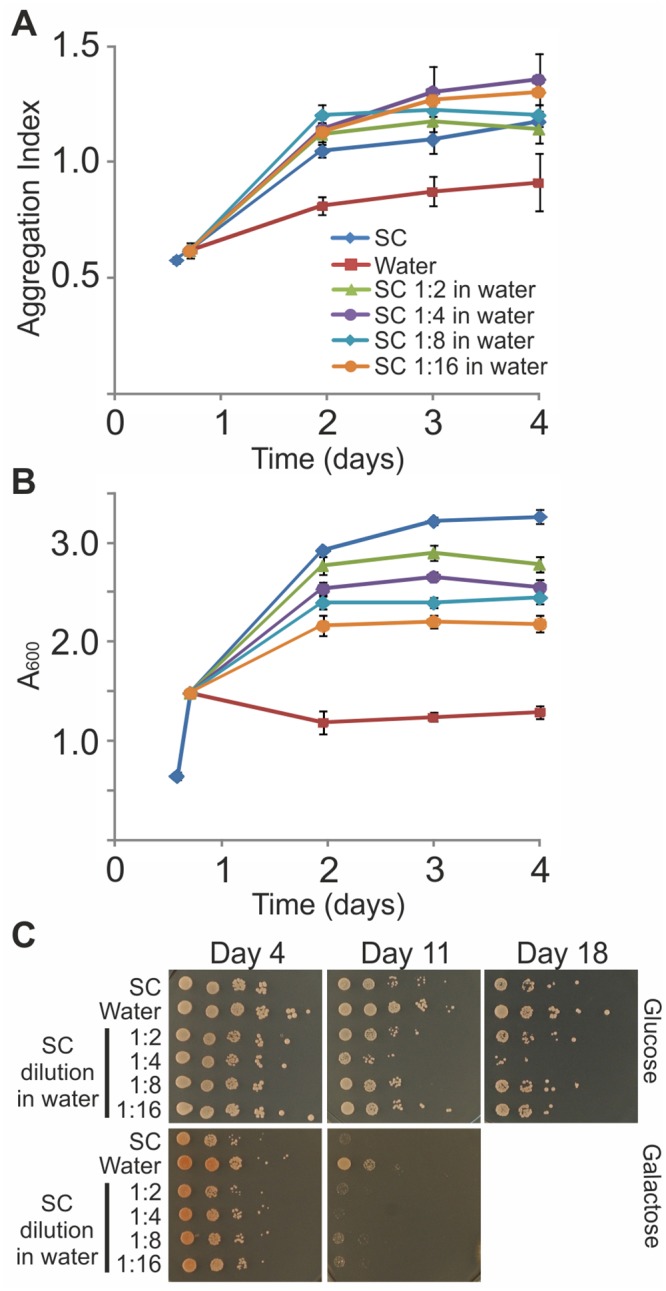
Effects of water-transfer on aggregation and toxicity cannot be reproduced by medium dilution. W303-1b cells expressing the 47Q were logarithmically grown in SC-Ura under galactose induction. After 17 hours, cells were either maintained in SC-Ura with galactose, transferred to water, or one volume of their SC medium was diluted with the indicated volumes of water, without changing the cell concentration. On the indicated days, (A) Aggregation Index was calculated, (B) growth curves were plotted (mean ± SE of 3 biological replicates and technical triplicates within each biological replicate), and (C) CFU viability was tested by spotting on glucose or galactose plates.

As water-transfer is considered 'extreme CR' [31], if its effect is merely due to nutrients depletion, stepwise dilution of media with water should cause a gradual change in aggregation, toxicity, and lifespan. To test this possibility, 47Q-expressing yeast was transferred to serial dilutions of SC medium in water. Cells grown for 17 hours in SC with galactose were either maintained in SC with galactose, transferred to pure water, or one volume of their SC medium was diluted with various volumes of pure water, without changing the cell concentration. Aggregation Index, growth and viability were followed (Fig 4). Clearly, diluting the medium in water up to 16 fold had no effect on 47Q aggregation (Fig 4A), despite the observed gradual decrease in biomass (Fig 4B). Moreover, the beneficial effect of water-transfer, which attenuated polyQ toxicity (Fig 2C), was not reproduced by diluting the medium (Fig 4C). Thus, the observed effects of water-transfer result neither from nutrients depletion *per se* nor from slowing down cell division. It is possible that ‘aging factors’ were secreted before medium dilution, and although their concentration decreased, they could still exert their effect, since a similar response in terms of protein aggregation and resistance to polyQ toxicity was observed in the 1:16 diluted medium and the non-diluted medium (Fig 4). We interpret these results to suggest that upon transfer to water a unique aging program is activated.

### Survival in water and its effects on aggregation require continuous protein synthesis and is supported by proteasomes

**Fig 5:**
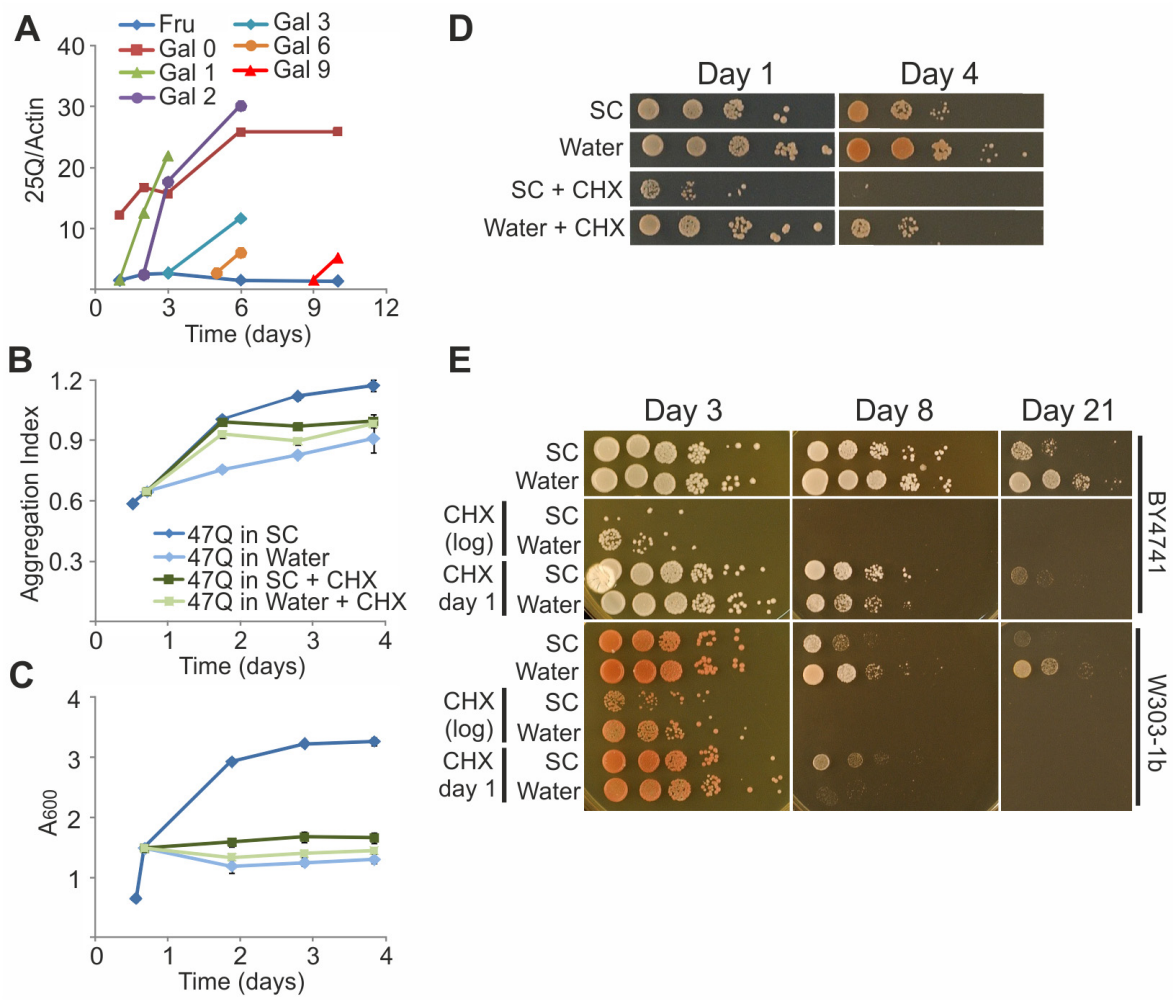
Survival in water and its effect on aggregation require continuous protein synthesis. (A) W303-1b cells expressing the 25Q were grown in SC-Ura supplemented with 2% fructose (Fru). Galactose (4%) was added either immediately (Gal 0) or on the indicated following days (Gal 1–9). On the indicated days afterwards, cells were collected and lysed, and proteins were dot-blotted onto nitrocellulose. Levels of 25Q and actin were determined by quantitative immunoblotting using rabbit anti-GFP and mouse anti-actin antibodies, respectively, as descried in Materials and Methods. (B-D) W303-1b cells expressing the 47Q were logarithmically grown in SC-Ura under galactose induction. After 18 hours, cells were either kept in SC-Ura with galactose or in SC-Ura with galactose supplemented with CHX (20 μg/ml), or transferred to water or to water supplemented with CHX. On the indicated days, (B) Aggregation Index was calculated, (C) growth curves were plotted (mean ± SE of 2 biological replicates and technical triplicates within each biological replicate), and (D) CFU viability was tested by spotting on YPD plates. (E) W303-1b or BY4741 cells, logarithmically grown (0.5–0.9 A_600_) in SC containing 2% glucose were either maintained in the same medium or transferred to water at logarithmic phase. Upon transfer (log) or on the following day (day 1), CHX (20 μg/ml) was added. Cells collected on the indicated days were tested for CFU viability on YPD plates. Shown is a representative experiment out of similar three.

Aging has been associated with declining capacity to synthesize proteins [35]. To assess whether aging yeast is capable of inducing genes and synthesize new proteins, cells harboring the short and non-toxic 25Q were maintained for up to 10 days in SC supplemented with fructose instead of glucose. While glucose inhibits 25Q induction, fructose does not interfere with induction by galactose. At different time points, galactose was added to the fructose-containing medium, to induce 25Q expression, which was monitored by immunoblotting. Clearly, the initial rates of the 25Q synthesis remained the same for the first 2 days following galactose addition, but decreased similarly between days 3 and 9 (Fig 5A). This may result from a global decline in the capacity to synthesize mRNA and/or proteins, or the increased fraction of dead cells. Hence, although protein synthesis in aging yeast is compromised, it is not abolished.

To test whether ongoing protein synthesis was required to attenuate 47Q aggregation upon water-transfer, cells were incubated with cycloheximide (CHX) while at logarithmic phase and then were transferred to water or maintained in SC with galactose. CHX is a direct inhibitor of protein biosynthesis that interferes with the translocation step, thus blocking translational elongation. We found that CHX eliminated the difference in 47Q aggregation between cells transferred to water or those maintained in SC, yielding Aggregation Index with intermediary values, i.e., lower than in cells maintained in SC but higher than in water-transferred cells (Fig 5B). As expected, yeast exposed to CHX in either SC or water stopped dividing (Fig 5C), and their survival decreased (Fig 5D). Together, these results indicate that protein synthesis is required not only for survival in water, as reported previously [11], but also for slowing the aging process in water, as manifested by antagonizing the stalled 47Q aggregation (Fig 5B). To test whether *de novo* protein synthesis was generally required for survival in water, CHX was added to untransformed BY4741 and W303-1b strains. We found that CHX was more toxic when added to logarithmic phase cells, in either SC or water, than when added at a later time point on day 1 (Fig 5E). Nevertheless, CHX still counteracted the beneficial effect of water-transfer when added on day 1 (Fig 5E) or even later, on day 9 (data not shown). These observations indicate that in order to survive in water, yeast must continuously synthesize new proteins not just at the initial stages of water-transfer. This ongoing protein synthesis may not support cell growth and division, but is probably required to either maintain core cellular processes indispensable for survival under extreme CR or to activate specific genetic programs that alter the cells’ metabolic state and adapt yeast to survive in water. With such meager resources, it is likely that water-transferred yeast synthesize only a limited set of the most essential proteins (see below).

**Fig 6:**
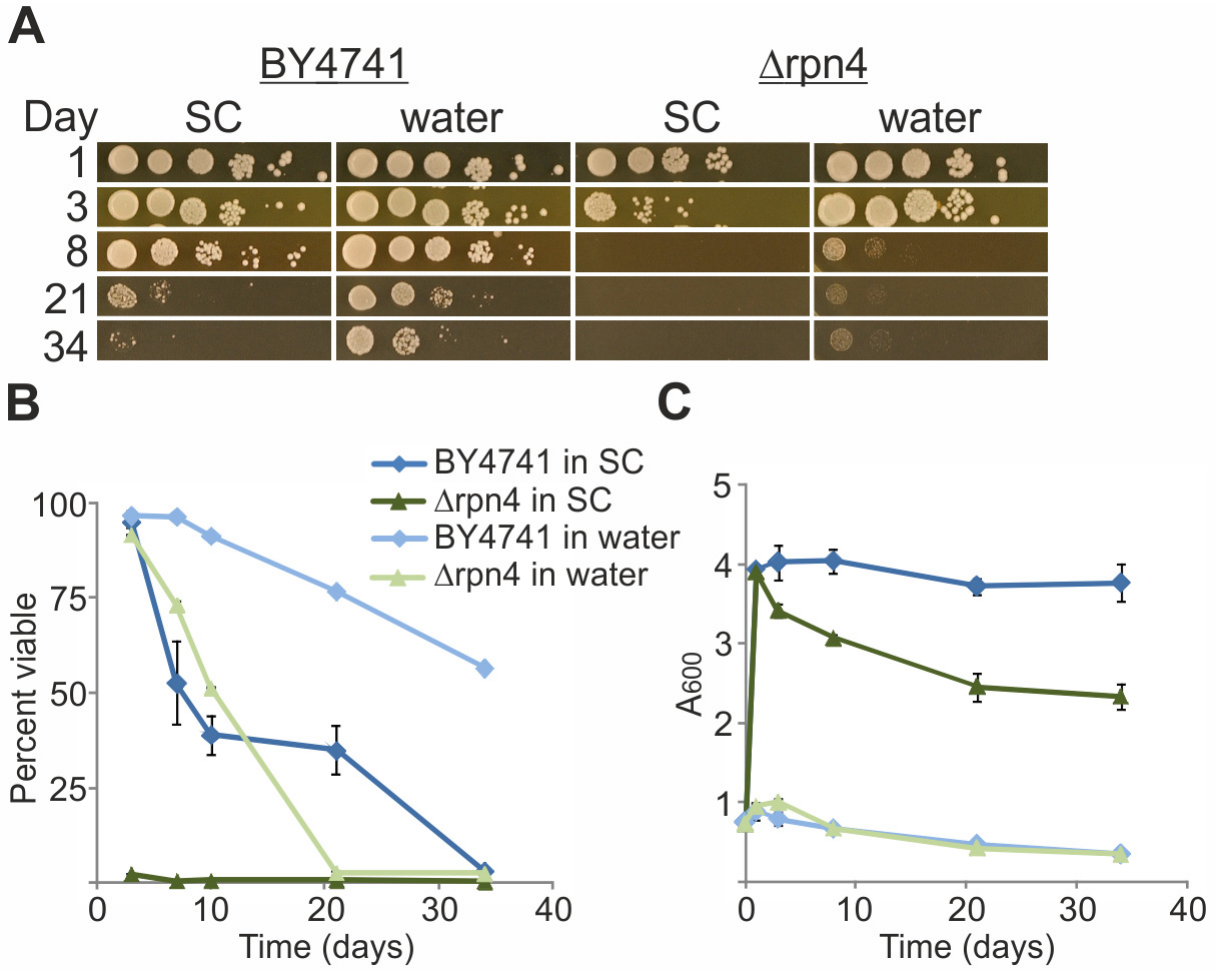
Without Rpn4, yeast survives poorly and is only partially rescued by water-transfer. BY4741 or Δ*rpn4* cells derived from them were grown in SC with 2% glucose. At 0.5–0.9 A_600_ (logarithmic growth) cells were either transferred to water or maintained in SC with glucose. On the indicated days (A) 10-fold serial dilutions (starting with 7.5x10^6^ cells) were spotted on YPD plates, and (B) viability was monitored by PI staining as described in Fig 1B. (C) Growth curves were plotted from 3 biological replicates and technical triplicates within each biological replicate. Data are presented as mean ± SE.

**Fig 7:**
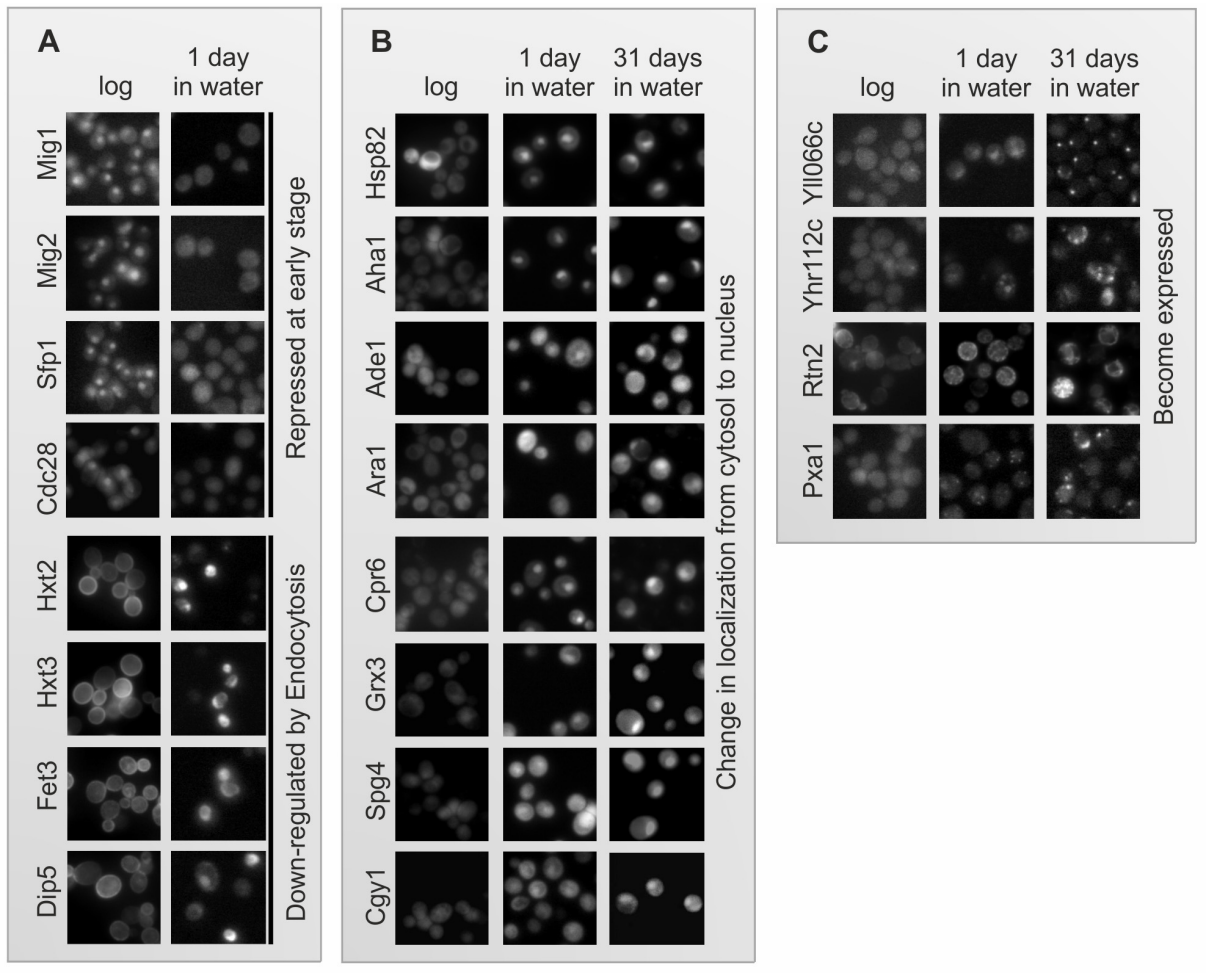
Single cell proteomics of water-transferred yeast uncovers dramatic changes in protein expression and localization. The MATa XXX-GFP::HIS3 library was grown over night on YPD agar plates and then transferred into 50 μl SC-His with 2% glucose in 384-well polystyrene flat-bottom plates, as described in Materials and Methods. Following transfer to water, over 5000 GFP-tagged yeast proteins were visualized during survival in water (after 1 day and 31 days). (A) Proteins that became repressed at an early stage and lost their fluorescent signal and proteins whose levels decreased as they were transported from the cell surface to the vacuolar lumen. (B) Proteins that retained their expression in water but moved from the cytosol to the nucleus. (C) Four proteins that were not expressed in cells growing logarithmically in SC but became expressed upon water transfer. For a complete list, see S2 Table

If *de novo* protein synthesis is indeed a prerequisite for the prolonged survival in water, the source of the required amino acids poses a conundrum. It was previously reported that autophagy is essential to survive in water [14]. Another potential source of free amino acids may be through degradation of cellular proteins in the ubiquitin-proteasome pathway, akin to muscle atrophy upon starvation [36]. To explore this possibility, we examined the ability of Δ*rpn4* cells to survive in water. Rpn4 is a major transcription factor that regulates proteasome levels [37]. The results in Fig 6 demonstrate that, relative to their wild-type BY4741 counterparts, Δ*rpn4* cells are far more susceptible to aging in SC medium and survive poorly upon water-transfer, indicating that proteasomes are critically required to allow survival in water. Also, we observed that several proteins, such as cell surface transporters, are targeted to the vacuole, likely for degradation (see Fig 7C, below). Thus, protein degradation by autophagy, by the vacuole and/or by the proteasome can be an adequate source for amino acids to allow *de novo* translation of proteins required for survival in water.

### A variety of proteins are induced, repressed or alter their intracellular localization upon water-transfer

To follow the intracellular fate of individual proteins upon water-transfer, over 5000 yeast proteins tagged with GFP at their C-terminus [25] were visualized during survival in water after one day and after 31 days. Even after one day in water, 27 proteins were downregulated, as indicated by the complete loss of their fluorescent signal, among them 6 bud neck proteins, one amino acids permease and 10 nuclear proteins (Fig 7A and S2 Table). This repression at an early stage of water-transfer suggested that the cells were going into energy preservation mode. To that end, it was interesting to note that mitochondrial proteins maintained their expression level even after 31 days in water, suggesting dependence on respiration. However, while the majority of proteins stopped being expressed after 31 days in water, a large number of proteins behaved differently (See S2 Table for a list of all changes). Eight proteins were removed from the cell surface by endocytosis and detected in the vacuole, among them 5 transporters and one bud neck protein (Fig 7A and S2 Table). Eleven proteins retained their expression but moved from the cytosol to the nucleus, while 312 proteins maintained their expression as well as localization even after 31 days in water (Fig 7B and S2 Table). Finally, four proteins were below detection in yeast grown logarithmically in SC, but were detected in water-transferred cells already after 1 day and were maintained for 31 days in water (Fig 7C and S2 Table). Proteins that became expressed in water-transferred yeast might be important for survival in water and should contribute to our understanding of this unique physiology (Fig 7C and S2 Table). Combined, it indicates that the survival in water is an active process that requires cells to adapt to this situation via a genetic program.

### Stress responses are compromised in aging yeast but maintained upon water-transfer

**Fig 8:**
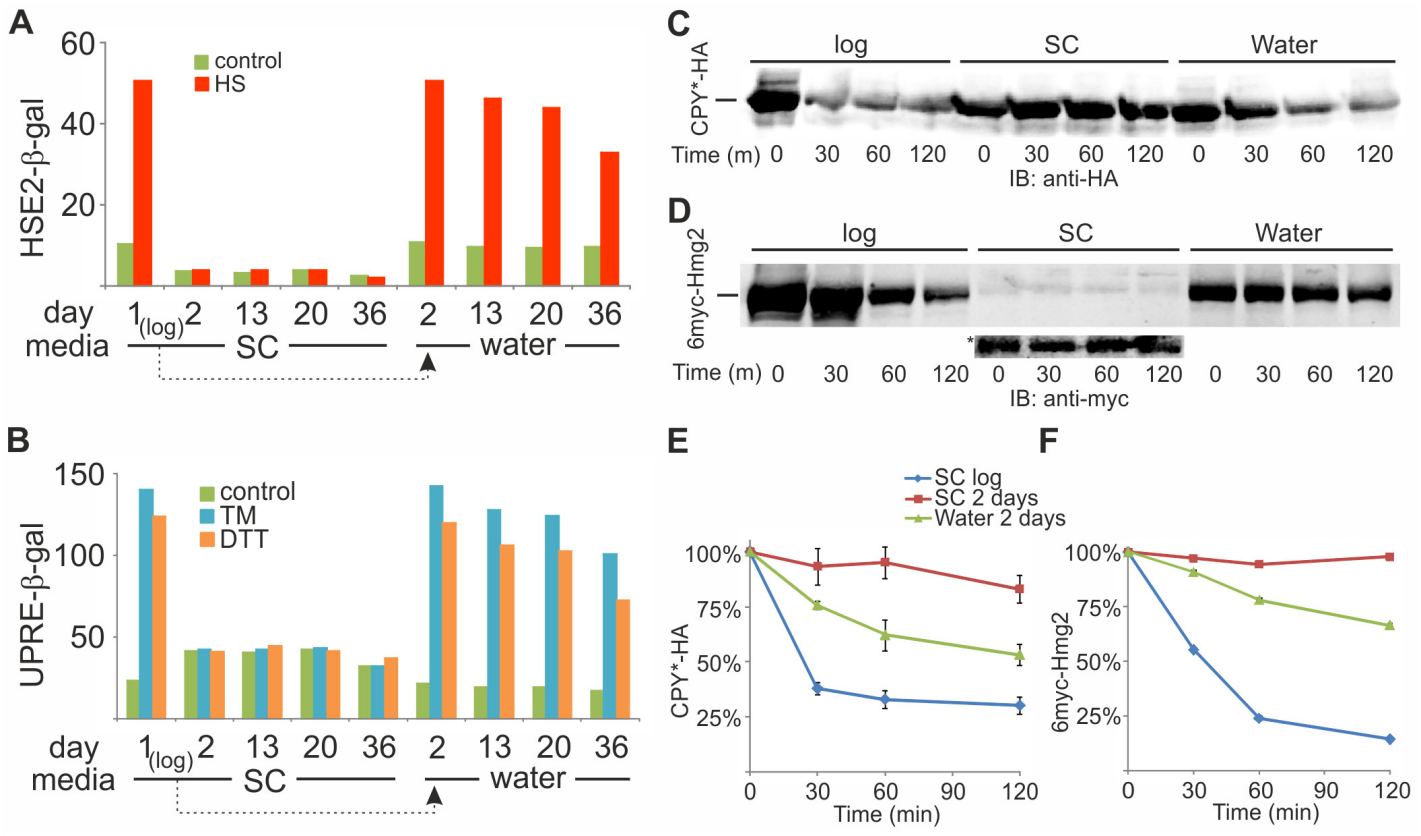
The HSR, UPR and ERAD are compromised in aging yeast but maintained upon water-transfer. Logarithmically growing (log) BY4741 cells harboring either HSE2-*LacZ* (A), UPRE-*LacZ* (B), CPY*-HA (C,E) or 6myc-Hmg2 (D,F) plasmids were either maintained in SC-Ura with glucose or transferred to water. On the indicated days, (A) cells were either subjected to a 20 minutes heat shock at 42°C (HS) or maintained for 20 minutes at 30°C (control); (B) cells were incubated for 1 hour without (control) or with either tunicamycin (TM; 5 μg/ml) or dithiothreitol (DTT; 6 mM). HSR (A) and UPR (B) were monitored as β-galactosidase specific activity. (C-F) Cells were subjected to CHX chase at log phase (log) or after 2 days in either SC-Ura with glucose or water. At the indicated time points after CHX addition, cells were collected and lysed and proteins were resolved by SDS-PAGE. CPY*-HA (C,E) and 6myc-Hmg2 (D,F) were detected by immunoblotting (IB) and quantified using Odyssey Infrared Imaging System. Asterisk, instrument’s settings were adjusted to view the low levels of 6myc-Hmg2. (E) Quantified data from 3 biological replicates are presented as mean ± SE. (F) A representative experiment out of 2 biological replicates.

We have recently shown that the heat shock response (HSR) declines dramatically as yeast age, and is completely lost in old yeast [29]. The HSR is orchestrated largely by Hsf1, a conserved transcription factor that regulates expression of many genes by binding to heat shock element (HSE) in their promoters [38]. As water-transfer appeared to slow the aging process, we monitored the HSR upon water-transfer of yeast expressing β-galactosidase under the HSE2 promoter [21]. To elicit the HSR, cells grown at 30°C were heat-shocked at 42°C for 20 minutes. In yeast that aged in SC, the HSR was completely lost within two days (Fig 8A and [29]), whereas upon water-transfer, the HSR was nearly fully preserved for over a month (Fig 8A).

We have recently reported that the HSR in yeast depends on an intact unfolded protein response (UPR), a link that is mediated by Sir2 [30]. Given this HSR-UPR link, we next followed the UPR along yeast aging, by measuring the activity of β-galactosidase expressed under the UPRE regulation. To elicit UPR, cells were exposed for 1 hr to either tunicamycin (TM) or dithiothreitol (DTT) [30]. Similarly to the HSR, the UPR also declined rapidly when yeast aged in SC, since after two days cells responded neither to TM nor to DTT (Fig 8B). However, water-transfer slowed this decline significantly, since TM- or DTT-elicited UPR remained robust even after one month in water (Fig 8B). Thus, two fundamental stress responses, the HSR and the UPR, which decline with yeast aging, are preserved upon water-transfer.

Dysfunction of the endoplasmic reticulum (ER)-associated protein degradation (ERAD), a key quality control process for eliminating aberrant proteins from the ER, has also been associated with aging [39], and ER chaperones have been shown to be oxidized with age [40]. The ERAD is tightly linked to the UPR, as the latter is activated when unfolded proteins within the ER exceed the capacity of the ER proteostasis machinery, and many ERAD-related factors are upregulated by the UPR [41–44]. With this UPR-ERAD interplay, and since the UPR was impaired in old yeast but maintained upon water-transfer (Fig 8B), it was of interest to explore whether the ERAD too followed the same pattern. We examined the degradation of two established ERAD substrates, the luminal mutant HA-tagged carboxy-peptidase Y (CPY*-HA), and the membrane myc-tagged 3-hydroxy-3-methylglutaryl coenzyme A reductase (6myc-Hmg2) [28]. When compared to the rapid degradation of both substrates in logarithmically growing cells, the ERAD of CPY*-HA and of 6myc-Hmg2 was fully halted in 2-days old yeast, but was largely preserved in water-transferred yeast (Fig 8C–8F). These results provide yet another indication that cellular proteostatic capacity decreases in aging yeast, but kept sustained to a large extent upon water-transfer, suggesting that the aging process is slowed in water-transferred yeast.

## Discussion

Current aging research focuses not only on lifespan extension, but also on maximizing healthspan–the period of vitality that is relatively free of aging-related diseases and physical impairment [45]. Aging research is mostly based on monitoring lifespan, which clearly reflects the aging process. However, cell death is an indirect measurement of the aging rate, as causes unrelated to aging may lead to cell death. For example, acetic acid, a byproduct of fermentation that was reported to act as an aging factor and to cause yeast cell death [12], had no effect on 47Q aggregation (data not shown). Thus, acetic acid represents a cell-extrinsic toxic agent that shortens lifespan without accelerating the aging process. Therefore, additional biomarkers for the aging process are in demand [2]. However, while lifespan can be easily monitored, readouts for the actual rate of the aging process are less obvious [2]. Indeed, neither CLS nor RLS address the yeast aging processes that precede cell death. Here we propose two novel readouts for the aging process that do not rely on lifespan measurements. These readouts also shed light on mechanisms underlying the longevity effect of water-transfer.

First, we confirm the observation that water-transfer greatly increases yeast lifespan (Fig 1A and 1B). We further extend this observation by demonstrating that yeast maintained in water retains characteristics of young cells, suggesting that this prolonged lifespan is the outcome of slowing the aging process. We have previously shown that processes underlying yeast aging start when cell replication slows, as reflected by growth curves approaching a plateau. At that time, the 47Q aging-reporter, which remains soluble in logarithmic phase cells, begins to aggregate [20]. Thus, 47Q aggregation serves as readout for the aging process, likely reflecting the diminished proteostasis (Fig 8). When yeast is transferred to water, 47Q aggregation stalls and the protein reaches lower aggregation levels (Fig 3A and 3C). In addition to the rate and extent of 47Q aggregation, we propose that the dramatic decline in three pathways of the proteostasis network, namely HSR, UPR, and ERAD (Fig 8), also serves as readout of the aging process in yeast. The timing at which these attributes decline parallels that of 47Q aggregation (Fig 3), attesting to the global effect of aging on deterioration of the proteostasis network, which, in turn, reflects changes in the proteome and especially the chaperome sub-network [46]. Such changes, which likely contribute to the aging-dependent 47Q aggregation, directly impact on protein aggregation in general, a hallmark of many aging-associated human diseases. It remains to be determined if the deterioration of the proteostasis network is the cause or consequence of aging, but it clearly correlates with this process. Remarkably, water-transfer markedly delays this decline, keeping robust HSR and UPR and functional ERAD for over a month (Fig 8). This slower deterioration of proteostasis likely results in the attenuated rate and extent of 47Q aggregation (Fig 3A and 3C). Thus, in general terms, our data demonstrate that transferring yeast to water increases their healthspan, as characteristics of young yeast are preserved for longer time.

Another beneficial aspect of water-transfer is the diminished toxicity of the aggregation-prone polyQ proteins (Fig 2C and 2D). This is probably the result of maintaining an intact proteostasis network. Moreover, with the limiting resources, low metabolism and growth arrest in water-transferred yeast, the burden of handling cellular damages and synthesizing new proteins is minimized. Interestingly, water-transferred cells that express either 47Q or 103Q show similar increased lifespan concomitant to reduced polyQ toxicity. Yet, the aggregation patterns of these polyQ proteins are distinct; the aging-dependent 47Q aggregation is slowed, whereas the aging-*in*dependent aggregation of 103Q proceeds (Fig 3). While the stalled 47Q aggregation probably reflects slower aging, the continued 103Q aggregation suggests that the intact proteostasis maintained in water-transferred yeast may protect the cells by handling this harmful protein, either sending it to degradation and/or depositing it in aggregates.

The effects of water-transfer cannot be simply regarded as an extreme case of CR [9] since these effects could not be replicated by diluting the media (Fig 4), even though responses to CR and water-transfer have been reported to activate overlapping pathways [47]. Moreover, stalling 47Q aggregation upon water-transfer is an active process(es) that requires continuous protein synthesis since 47Q similarly aggregates in rich medium or in water once protein translation is blocked (Fig 5B). This active process likely relates to the strategy yeast employs to survive in water, which totally depends on ongoing translation (Fig 5E), probably of a distinct set of proteins.

The significance of protein synthesis for survival in water is also supported by our screen of the GFP-tagged library of yeast proteins (Fig 7). As expected, the expression of 27 proteins is lost immediately upon water-transfer (Fig 7A and S2 Table) and the vast majority of proteins stop being expressed at a later stage and become undetectable by 31 days. However, there are at least four proteins whose expression is upregulated in water-transferred cells (Fig 7C and S2 Table). Whether and how these proteins support survival under such poor conditions is yet to be investigated. Moreover, 81 proteins change their localization upon water transfer (Fig 7B and S2 Table). As ongoing protein translation relies on supply of amino acids, which are clearly absent from water, it is intriguing that six cell surface transporters are targeted to the vacuole, likely for degradation (Fig 7C), and that proteasomes are also required for survival in water (Fig 6). Hence, protein degradation by the vacuole, by the proteasome, as well as by autophagy [14], adequately provides the amino acids necessary for *de novo* translation of proteins that is critical for survival in water. Combined, our data indicate that the survival in water is an active process in which a genetic program enables cells to adapt to this extreme environment.

The novel aging readouts described in the current study are all delayed in water-transferred yeast. Thus, we suggest that the rate of the aging process is slowed in water, which, in turn, results in prolonged longevity. This unique phenomenon provides insights into genes and cellular processes that operate in aging. It remains to be determined if the extended lifespan and healthspan of water-transferred yeast is associated with low metabolism and whether it is accompanied by repression of ‘aging genes’ and/or if and how genes that are induced upon water-transfer contribute to yeast ‘youthfulness’. It is plausible that manipulating the activities of such genes would mimic the extended lifespan observed upon water-transfer. For instance, sporulation-related genes are activated when diploid cells are starved for nitrogen in the presence of a poor carbon source, and the spores remain viable for many years, possibly centuries [48]. Here we show that while the expression of the vast majority of proteins is turned down by 31 days in water, probably reflecting an energy preservation mode, 312 proteins, such as mitochondrial proteins, remain constantly expressed even after 31 days in water (S2 Table), suggesting the importance of respiration. Conversely, specific transcriptional repressors (e.g., Mig1 and Mig2; S2 Table), which are involved in glucose repression, are downregulated very early on. Similarly, six transporters on the plasma membrane get internalized, probably reflecting their redundancy in water, and bud neck and spindle assembly proteins stop being expressed as cells stop dividing. Whether and how proteins whose expression and/or localization are affected upon water-transfer contribute to the extended lifespan and slow aging is yet to be determined. The 47Q aging biomarker can serve as a valuable tool for uncovering genes related to slowing aging and survival in water. Further characterization of the water-transfer effect and understanding how water-transfer slows aging in yeast may provide new insights into the aging process in humans and how it can be slowed to prolong healthspan.

## Supporting Information

S1 Table Composition of SC media. For details see Materials and Methods and individual figures.
(DOCX)

S2 Table List of proteins affected by water-transfer. For details see Materials and Methods and Fig 7.
(XLSX)
