# Supplementary material for: Water-Transfer Slows Aging in Saccharomyces cerevisiae
Source: PLoS One. 2016 Feb 10;11(2):e0148650. doi: 10.1371/journal.pone.0148650 (PMC4749178; doi:10.1371/journal.pone.0148650)
Supplement: S1 Table — For details see Materials and Methods and individual figures. (DOCX) [file pone.0148650.s001.docx]

| **Component** | **Type 1**  **(mg/lit)** | **Type 2**  **(mg/lit)** |
| --- | --- | --- |
| Use | Plasmids: polyQ;  HSE-LacZ; UPRE-LacZ;  6myc-Hmg2; CPY*-HA | Screening the MATa XXX-GFP::HIS3 library |
| Yeast Nitrogen Base with  Ammonium Sulfate | 6700 | 6700 |
| Isoleucine | 30 | 30 |
| Valine | 130 | 150 |
| Adenine | 20 | 40 |
| Arginine | 20 | 20 |
| Histidine | 20 | NONE |
| Leucine | 100 | 100 |
| Lysine | 30 | 30 |
| Methionine | 20 | 20 |
| Phenylalanine | 50 | 50 |
| Threonine | 200 | 200 |
| Tryptophan | 20 | 40 |
| Tyrosine | 30 | 30 |
| Uracil | NONE | 20 |
| Glutamic | 87 | - |
| Aspartic | 87 | - |
| Serine | 348 | - |
